# Unraveling the role of quorum sensing-dependent metabolic homeostasis of the activated methyl cycle in a cooperative population of *Burkholderia glumae*

**DOI:** 10.1038/s41598-019-47460-6

**Published:** 2019-07-30

**Authors:** Yongsung Kang, Hongsup Kim, Eunhye Goo, Hyesung Jeong, Jae Hyung An, Ingyu Hwang

**Affiliations:** Department of Agricultural Biotechnology, Seoul National University, Seoul, 08826 Republic of Korea

**Keywords:** Bacterial physiology, Bacterial genetics

## Abstract

The activated methyl cycle (AMC) is responsible for the generation of *S*-adenosylmethionine (SAM), which is a substrate of *N*-acylhomoserine lactone (AHL) synthases. However, it is unknown whether AHL-mediated quorum sensing (QS) plays a role in the metabolic flux of the AMC to ensure cell density-dependent biosynthesis of AHL in cooperative populations. Here we show that QS controls metabolic homeostasis of the AMC critical for AHL biosynthesis and cellular methylation in *Burkholderia glumae*, the causal agent of rice panicle blight. Activation of genes encoding SAM-dependent methyltransferases, *S*-adenosylhomocysteine (SAH) hydrolase, and methionine synthases involved in the AMC by QS is essential for maintaining the optimal concentrations of methionine, SAM, and SAH required for bacterial cooperativity as cell density increases. Thus, the absence of QS perturbed metabolic homeostasis of the AMC and caused pleiotropic phenotypes in *B*. *glumae*. A null mutation in the SAH hydrolase gene negatively affected AHL and ATP biosynthesis and the activity of SAM-dependent methyltransferases including ToxA, which is responsible for the biosynthesis of a key virulence factor toxoflavin in *B*. *glumae*. These results indicate that QS controls metabolic flux of the AMC to secure the biosynthesis of AHL and cellular methylation in a cooperative population.

## Introduction

Most *N*-acylhomoserine lactone (AHL) synthases use *S*-adenosylmethionine (SAM) and acyl–acyl carrier proteins (acyl-ACP) as substrates^1^. SAM is a major methyl donor in cells but acts as the donor of the lactone moiety in AHL biosynthesis^1,2^. The activated methyl cycle (AMC) is an important metabolic pathway responsible for producing SAM and for *de novo* methionine biosynthesis^3^. In the AMC, a SAM synthase encoded by *metK* catalyzes the formation of SAM from methionine and adenine triphosphate (ATP)^4^. Upon methyl transfer from SAM to nucleic acids, proteins, or other compounds by SAM-dependent methyltransferases, SAM is converted into *S*-adenosylhomocysteine (SAH)^3,5^. SAH is converted to homocysteine by SAH hydrolase encoded by *ahcY* in AHL-producing bacteria^6^ or by a two-step reaction of Pfs and LuxS in autoinducer-2 (AI-2) producers^7,8^. Thus, the AMC and biosynthesis of 4,5-dihydroxy-2,3-pentanedione, an AI-2 precursor, are directly coupled^7–9^. Methylenetetrahydrofolate reductase (MetF) converts 5,10-methylenetetrahydrofolate into 5-methytetrahydrofolate, which serves as a methyl donor to homocysteine via either vitamin B_12_-dependent or -independent methyltransferase, designated MetH or MetE, respectively, in *Escherichia coli*^10,11^. It is important to understand how control of the AMC affects biogenesis of quorum signals in quorum sensing (QS) bacteria.

Our understanding of AHL biosynthesis has mainly focused on the biochemistry of AHL synthesis and the regulation of AHL biosynthetic genes^12,13^. Exponential biosynthesis of AHL might require systematic control of the AMC to provide sufficient SAM as a substrate along with acyl-ACP. Considering that both SAM-dependent methyltransferases and AHL synthases use SAM as a substrate, it is important to determine whether QS controls the expression of SAM-dependent methyltransferase genes in AHL producing QS bacteria. Despite the important role that SAH hydrolase plays in the detoxification of SAH in cell metabolism^14^, it is not known whether QS affects expression of *ahcY* in AHL-producing QS bacteria. Furthermore, the importance of the *in vivo* metabolic balance between SAM and SAH for AHL biosynthesis has not been addressed in AHL producers.

To explore how the metabolic systems of the AMC in individuals have evolved to support successful AHL biosynthesis for cooperative populations, we used *Burkholderia glumae* as a model organism. This species, which is the pathogen that causes rice panicle blight, has a relatively easy-to-handle QS system with a single LuxR/LuxI type^15^. In *B*. *glumae*, TofI directs biosynthesis of *N*-octanoyl-homoserine lactone (C8-HSL), the cognate receptor of which is TofR^15^. A complex of C8-HSL and TofR then activates expression of an IclR-type transcriptional regulator, QsmR^16^. In this study, we addressed two major questions regarding the role of the AMC for sustainable cooperativity in *B*. *glumae*. First, we determined whether QS influences the expression of genes involved in the AMC, and the metabolic flux of the AMC as cell density increases. Second, we evaluated the importance of metabolic balance of the AMC for social activities of *B*. *glumae*. We found that the metabolic homeostasis of the AMC depends on QS and plays critical roles in AHL biosynthesis, cellular methylation, ATP biosynthesis, and virulence of *B*. *glumae*.

## Results

### Regulation of SAM-dependent methyltransferase genes by QS

**Figure 1:**
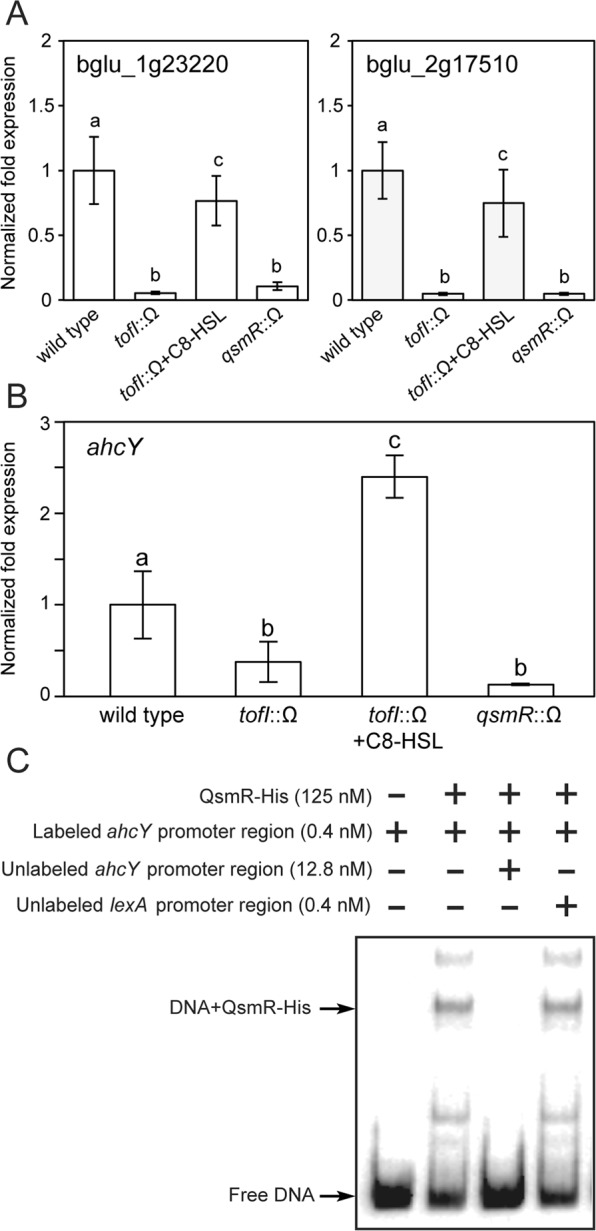
Activation of S-adenosylmethionine (SAM)-dependent methyltransferase and *ahcY* genes by quorum-sensing (QS) in *B*. *glumae*. (**A**) Expression levels of SAM-dependent methyltransferase genes and (**B**) *ahcY* in *B*. *glumae* BGR1 (wild type), BGS2 (BGR1 *tofI*::Ω), and BGS9 (BGR1 *qsmR*::Ω). Gene expression levels were quantified by quantitative real-time PCR (qRT-PCR) after 10 h of incubation with three biological replicates. The letters (a, b, and c) above each mean represent statistical significance based on ANOVA/Tukey’s correction for multiple comparisons. A value of p < 0.05 represents a significant difference among strains. (**C**) Binding of QsmR-His to the putative promoter region of *ahcY*. Gel mobility shift assays show that purified QsmR-His binds to the DNA fragment carrying putative promoter region of *ahcY*.

Based on our previous RNA sequencing results of the wild type strain BGR1 and two QS mutants, BGS2 (BGR1 *tofI*::Ω) and BGS9 (BGR1 *qsmR*::Ω), we identified three out of eight SAM-dependent methyltransferase genes, including the previously known *toxA* (bglu_2g06400) gene responsible for toxoflavin biosynthesis, as potentially being regulated by QS (Supplementary Table 1). To validate this possibility, we analyzed the expression of each gene by quantitative real-time PCR (qRT-PCR). We found that expression of two SAM-dependent methyltransferase genes, bglu_1g23220 and bglu_2g17510, was significantly lower in BGS2 and BGS9 than in the wild type, but expression levels in the *tofI* mutant BGS2 were recovered to wild-type levels by the addition of 1 μM of exogenous C8-HSL (Fig. 1A). These results confirm that expression of three SAM-dependent methyltransferase genes (including *toxA*) is activated by QS in *B*. *glumae*.

### QsmR activates expression of ahcY

As our previous RNA sequencing results of the wild type strain BGR1 and two QS mutants, BGS2 (BGR1 *tofI*::Ω) and BGS9 (BGR1 *qsmR*::Ω), indicated that QS might regulate the expression of *ahcY* (bglu_1g01990), we performed qRT-PCR analysis of *ahcY* in BGR1, BGS2, and BGS9 to confirm these results. Expression of *ahcY* was approximately 2–3 times higher in the wild type than in the two QS mutants (Fig. 1B). Exogenous addition of 1 μM C8-HSL to the *tofI* mutant BGS2 rescued expression of *ahcY* (Fig. 1B). An electrophoretic mobility shift assay with purified QsmR-His and the putative promoter region of *ahcY* confirmed that QsmR directly activated expression of *ahcY* (Fig. 1C).

### Regulation of *de novo* methionine biosynthesis by QS

**Figure 2:**
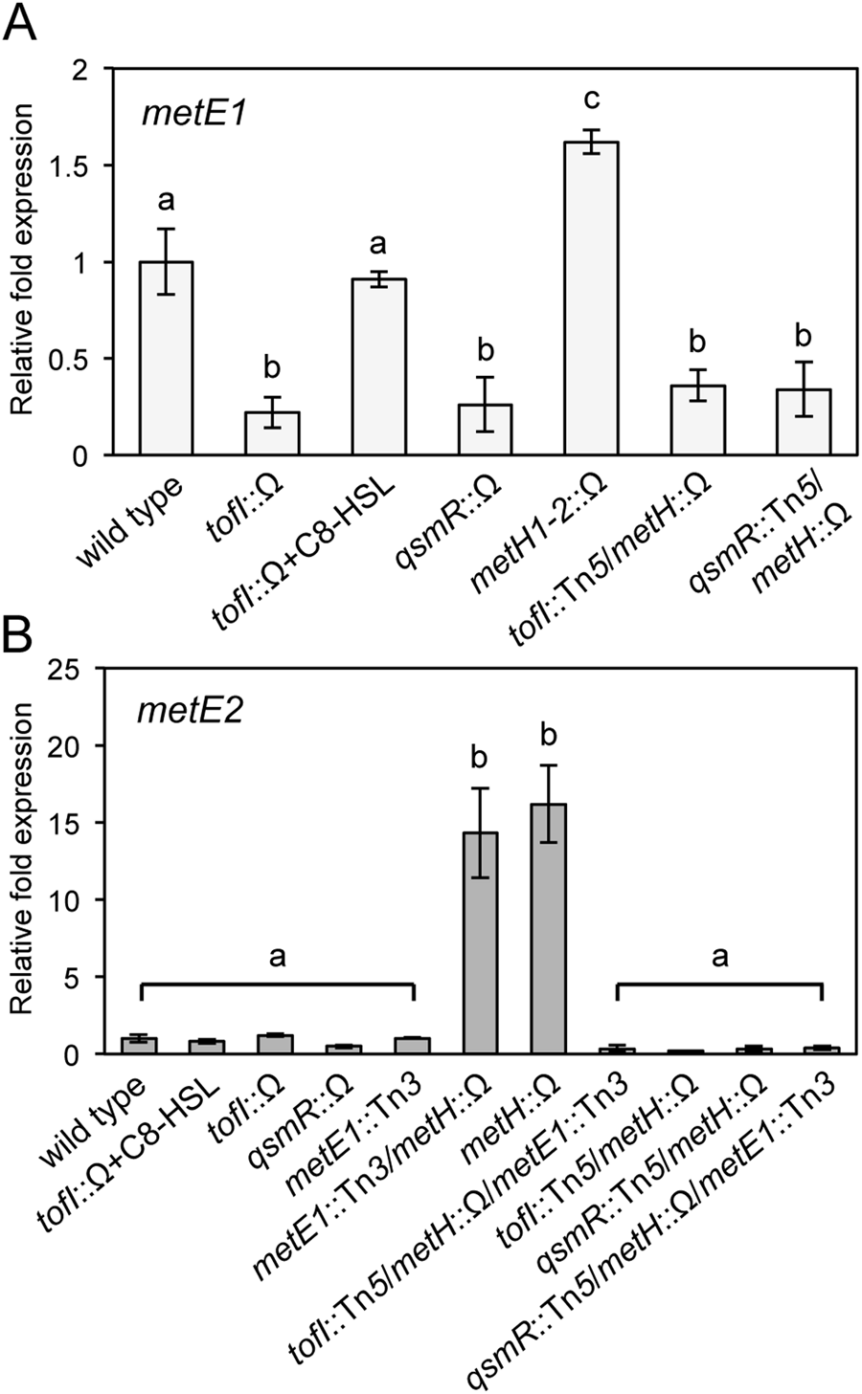
Regulation of *metE1* and *metE2* expression in *B*. *glumae* BGR1. (**A**) Expression levels of *metE1* in the wild type, QS mutants, and *metH* mutants. Gene expression levels were quantified by qRT-PCR after 10 h of incubation, with three biological replicates. (**B**) Effect of the presence of *metH* on the expression of *metE2*. Expression levels of the *metE2* gene in the wild type, QS mutants, *metE1* mutant, and *metH* mutant, as measured by qRT-PCR using a *metE2*-specific primer. The following strains were used in the experiments: BGR1 (wild type), BGS2 (BGR1 *tofI*::Ω), BGS9 (BGR1 *qsmR*::Ω), BMH1 (BGR1 *metH1-2*::Ω), BME1 (BGR1 *metE1*::Tn*3-gusA36*), BMEH1 (BGR1 *metE1*::Tn*3-gusA36*/*metH1-2*::Ω), BMH2 (BGR1 *tofI*::Tn5/*metH1-2*::Ω), BMEH2 (BGR1 *tofI*::Tn5/*metH1-2*::Ω/*metE1*::Tn*3-gusA36*), BMH9 (BGR1 *qsmR*::Tn5/*metH1-2*::Ω), and BMEH9 (BGR1 *qsmR*::Tn5/*metH1-2*::Ω/*metE1*::Tn*3-gusA36*). Error bars represent the error ranges of the experiments performed in triplicate. The letters (a, b, and c) above each mean represent statistical significance based on ANOVA/Tukey’s correction for multiple comparisons. A value of p < 0.05 represents significant differences among strains.

There are two copies each of *metE* and *metH* genes in *B*. *glumae*: *metE1* (bglu_1g08430) and *metE2* (bglu_2g04930), and *metH1* (bglu_1g32280) and *metH2* (bglu_1g32290). MetE1 and MetE2 share 53% identity (Supplementary Fig. 1). The proteins produced by the two respective MetH genes (MetH1; 38.2 KDa and MetH2; 99.6 KDa) aligned to the N-terminus and C-terminus of *E*. *coli* MetH, respectively, with 63% identity (Supplementary Fig. 1). Previous RNAseq analysis of *metE1* expression indicated its QS-dependency, and we confirmed this by qRT-PCR analysis. Expression of *metE1* was lower in the two QS mutants than in the wild type BGR1 but was recovered to the wild-type level by exogenous addition of 1 µM C8-HSL to BGS2 (Fig. 2A). Expression of *metE2* was very low in the wild type BGR1 and in the two QS mutants (Fig. 2B). To determine whether functions of MetH1, MetH2, or MetE1 influence expression of *metE2*, we generated an internal deletion mutant of *metH1* and *metH2* and an insertional mutant of *metE1* (Supplementary Fig. 2) and estimated expression levels of *metE2* in BMH1 (BGR1 *metH1-2*::Ω), BME1 (BGR1 *metE1*::Tn3*-gusA*3*6*), and BEM12 (BGR1 *metE1*::Tn3*-gusA*3*6/metH1-2*::Ω). In BMH1, expression of *metE2* was much higher than in the wild type, increasing in a QS-dependent manner (Fig. 2B). The absence or presence of MetE1 had no effect on the expression of *metE2* (Fig. 2B). These results indicated that either the level or activity of MetH1 and MetH2 affect the expression of *metE2*.

To determine whether TofR and/or QsmR directly activate the expression of *metE1*, we purified TofR-His and QsmR-His and found that both proteins individually bound to the putative promoter region of *metE1* (Supplementary Fig. 3A). When TofR-His and QsmR-His were applied together to the putative promoter region of *metE1*, a larger molecule (complex) was produced than that observed in response to treatment with TofR-His or QsmR-His alone (Supplementary Fig. 3B). These results indicate that TofR and QsmR bind simultaneously to the regulatory region of *metE1*. The implications of this for *metE1* expression are unknown.

To determine the contribution of the different *metH* and *metE* homologs to methionine biosynthesis in *B*. *glumae*, we monitored the growth of BME1 (BGR1 *metE1*::Tn*3-gusA36*), BME12 (BGR1 *metE1*::Tn*3-gusA36/metE2-metR2*::Gm^r^), BMH1 (BGR1 *metH1-2*::Ω), and *E*. *coli* JW3805, a *metE* deletion mutant of *E*. *coli* in M9 minimal medium. Although all strains of *B*. *glumae* grew in M9 minimal medium, *E*. *coli* JW3805 did not, requiring vitamin B_12_ for growth (Supplementary Fig. 4).

### Metabolic perturbation of the AMC in QS mutants

**Figure 3:**
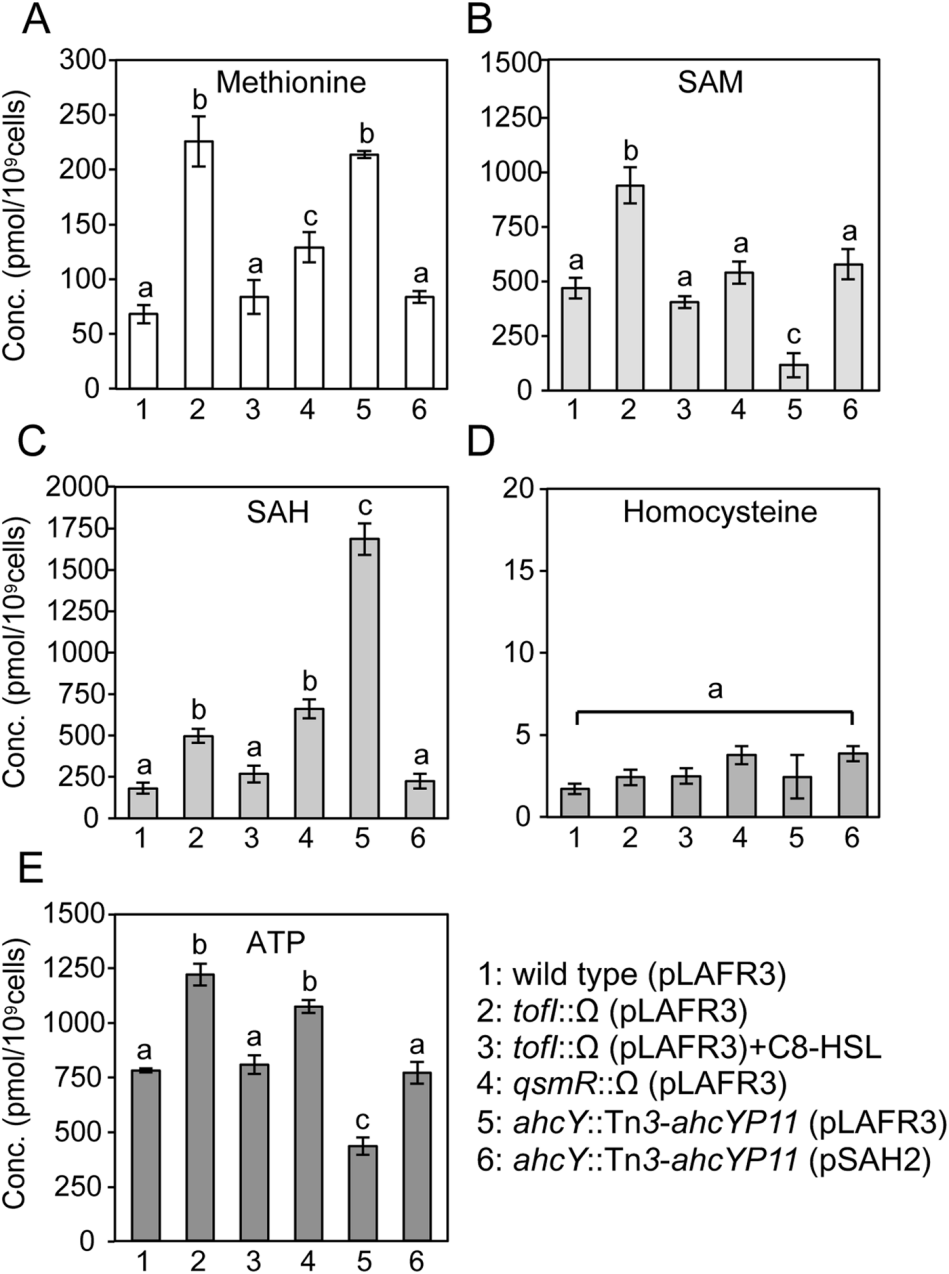
Cellular concentrations (conc.) of activated methyl cycle (AMC)-related compounds in *B*. *glumae* strains. Strains used for the analysis are labeled as follows: 1, BGR1 (wild type/pLAFR3); 2, BGS2 (BGR1 *tofI*::Ω/pLAFR3); 3, BGS2 (pLAFR3) plus 1 µM C8-HSL; 4, BGS9 (BGR1 *qsmR*::Ω/pLAFR3); 5, BAH2 (BGR1 *ahcY*::Tn*3-ahcYP11*/pLAFR3); 6, BAH2 (pSAH2). The plasmid pSAH2 carries a 3.3-kb fragment with a putative promoter region and *ahcY* gene in pLAFR3. Intracellular concentrations of (**A**) methionine, (**B**) SAM, (**C**) *S*-adenosylhomocysteine (SAH), (**D**) homocysteine, and (**E**) adenine triphosphate (ATP) in wild type, QS mutants, and *ahcY* mutants. Metabolite levels were measured after 10 h of incubation, with three biological replicates. Values are expressed as picomoles per 1 × 10^9^ cells. The letters (a, b, and c) above each mean represent statistical significance based on ANOVA/Tukey’s correction for multiple comparisons. A value of p < 0.05 represents significant differences among strains.

Since QS positively controlled the AMC, we determined intracellular concentrations of SAM, SAH, homocysteine, and methionine in BGR1, BGS2, and BGS9, in the late exponential growth stage. Concentrations of methionine, SAM, and SAH were significantly higher in BGS2 and BGS9 than in BGR1, whereas homocysteine levels were relatively constant in all three strains (Fig. 3A–D). Exogenous addition of 1 μM C8-HSL to BGS2 restored methionine, SAM, and SAH concentrations to wild-type levels (Fig. 3A–C). These results indicate that QS plays an important role in maintaining optimal concentrations of each AMC-related compound at high cell densities.

### Co-transcription of *ahcY* and *metF* genes

**Figure 4:**
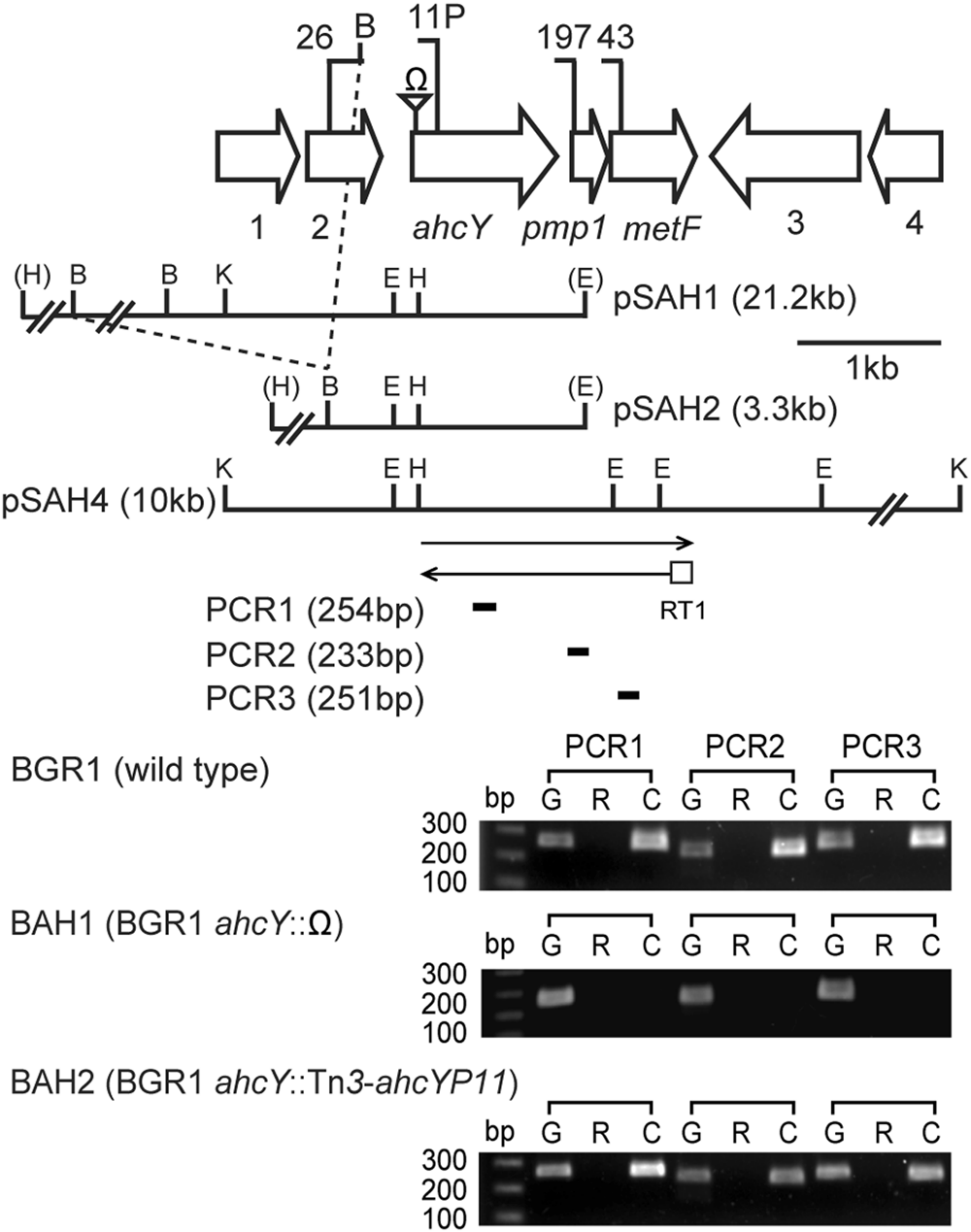
Genetic organization and mutant construction of genes in the *ahcY* locus in *B*. *glumae* BGR1. Schematic organization of the *ahcY* locus: 1, bglu_1g01970; 2, bglu_1g01980; *ahcY*, bglu_1g01990; *pmp1*, bglu_1g02000; *metF*, bglu_1g02010; 3, bglu_1g02020; 4, bglu_1g02030 (GenBank accession numbers: CP001503–CP001508). Arrows with arrowheads represent the direction and length of cDNA. Vertical bars in the map denote the positions and orientations of Tn*3-gusA* insertions; vertical bar with inverted triangle indicates position of the Ω cassette insertion; “P” indicates the promoter region of the *ahcY* gene. The restriction enzyme sites are indicated as follows: E, EcoRI; B, BamHI; H, HindIII; K, KpnI. Enzyme sites from the vector are shown in parentheses. The short thick bars below the RT arrows indicate PCR products from the corresponding RT reactions. The expected sizes of the PCR products are indicated in parentheses beside each numbered PCR. Lane G: PCR products from chromosomal DNA as a template; lane R: PCR products from total RNA; lane C: PCR products from cDNA.

The genetic organization of the *ahcY*-*metF* region varies among bacterial taxa (Supplementary Fig. 5). In *B*. *glumae*, three genes, including *ahcY*, *metF* (bglu_1g02010), and *pmp1* (bglu_1g02000), which encodes a predicted membrane protein with unknown function, clustered together (Fig. 4). A similar gene organization was observed in *Pseudomonas aeruginosa* PAO1, except that *pmp1* was not found between *ahcY* and *metF* (Supplementary Fig. 5). In *Agrobacterium tumefaciens* C58 and *A*. *radiobacter* K84, *ahcY* and *metF* were not clustered (Supplementary Fig. 5). Neither *ahcY* nor *pmp1* were found in *E*. *coli*, *Salmonella*, *Vibrio*, or *Mycobacterium* species (Supplementary Fig. 5).

To determine whether *ahcY* is co-transcribed with *pmp1* and *metF* in *B*. *glumae*, we performed reverse transcription-PCR (RT-PCR) analysis using cDNA synthesized from wild-type (BGR1) mRNA with specific primers (Supplementary Table 2). We found that *ahcY*, *pmp1*, and *metF* were co-transcribed (Fig. 4). An insertion of the Ω fragment in *ahcY* had polar effects, as confirmed by RT-PCR and growth of the mutant in M9 minimal medium (Supplementary Fig. 6). Although disruption of *pmp1* or *metF* has a strong negative influence on growth in minimal medium, insertion into *ahcY* still allows survival with very limited growth (Supplementary Fig. 6). It is possible that there is also a promoter immediately upstream of *pmp1*-*metF* which allows sufficient expression of these genes to allow growth of the mutant with omega insertion in *ahcY*. To avoid polarity caused by the insertion of the Ω fragment in *ahcY*, we constructed a non-polar mutant of *ahcY* by inserting a modified Tn*3*-*gusA* possessing a putative *ahcY* promoter region, Tn*3*-*ahcYP11*, in *ahcY*, resulting in BAH2 (BGR1 *ahcY*::Tn*3*-*ahcYP11*; Fig. 4). The non-polarity of BAH2 was confirmed by RT-PCR (Fig. 4) and growth in M9 minimal medium (Supplementary Fig. 7). Growth of BAH2 was retarded but was recovered by pSAH2 possessing a 3.3-kb DNA fragment that included *ahcY* along with its putative promoter region in pLAFR3 (Supplementary Fig. 7).

### Imbalance of SAM and SAH in the *ahcY* mutant negatively affected biosynthesis of C8-HSL and toxoflavin

**Figure 5:**
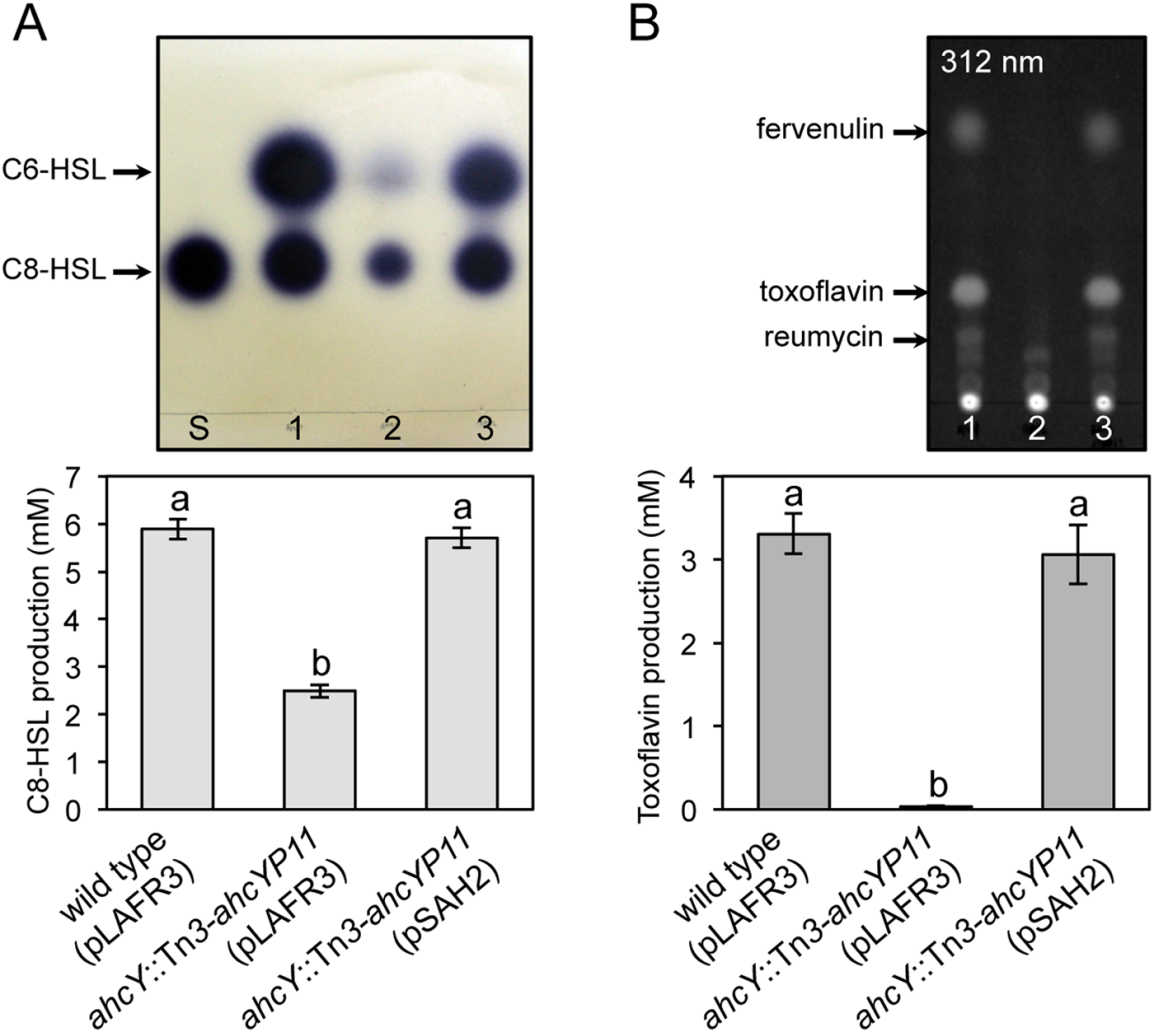
Inhibition of autoinducer and toxoflavin production in the *ahcY* mutant. (**A**) Thin-layer chromatography (TLC) of ethyl acetate extracts from the culture filtrates of *B*. *glumae* strains to measure autoinducer production (upper panel) and quantitative analysis of C8-HSL production in BGR1, BAH2 (pLAFR3), and BAH2 (pSAH2) (bottom panel). (**B**) TLC of chloroform extracts from the culture filtrates of *B*. *glumae* strains for analysis of toxoflavin production (upper panel) and quantitative analysis of toxoflavin production in the strains (bottom panel). The toxoflavin produced was detected under UV light at 312 nm. S, Standard C8-HSL 1 µM; 1, BGR1 (wild type/pLAFR3); 2, BAH2 (BGR1 *ahcY*::Tn*3-ahcYP11*/pLAFR3); 3, BAH2 (pSAH2). The error bars represent the error ranges of the experiments performed in triplicate. The letters (a and b) above each mean represent statistical significance based on ANOVA/Tukey’s correction for multiple comparisons. A value of p < 0.05 represents a significant difference among strains.

To determine how a non-polar mutation in *ahcY* would affect cellular concentrations of methionine, SAM, SAH, homocysteine, and ATP, we measured the levels of each compound in the *ahcY* mutant BAH2. Concentrations of methionine and SAH were significantly higher in BAH2 than in the wild type BGR1, but SAM concentrations were significantly lower in BAH2 than in BGR1 (Fig. 3B,C). Homocysteine concentrations did not differ significantly in BAH2 compared to the wild type (Fig. 3D). The low concentration of SAM in BAH2 was not due to low expression of *metK* at transcription or translational levels (Supplementary Fig. 8) but was associated with lower levels of ATP for SAM synthesis (Fig. 3E). Genetic complementation of BAH2 with pSAH2 containing *ahcY* restored concentrations of each compound to wild-type levels (Fig. 3A–E). These results led us to investigate whether an imbalance of SAM and SAH in BAH2, wherein SAH levels were high and SAM levels were low, would affect the mutant’s ability to produce C8-HSL and toxoflavin. According to a quantitative analysis of toxoflavin and autoinducer production performed by thin-layer chromatography (TLC) from the culture filtrates of *B*. *glumae* strains (Fig. 5A,B), the *ahcY* mutant BAH2 produced approximately 3 times less C8-HSL than did the wild type, but C8-HSL production was recovered to the wild-type level by genetic complementation with pSAH2 (Fig. 5A). Toxoflavin production assays showed that BAH2 did not produce detectable amounts of toxoflavin, whereas BGR1 and pSAH2-complemented BAH2 (BGR1 *ahcY*::Tn*3*-*ahcYP11*/pSAH2) did (Fig. 5B). These results indicate that the presence of excess SAH due to a lack of *ahcY* inhibited TofI and ToxA activities *in vivo*.

**Figure 6:**
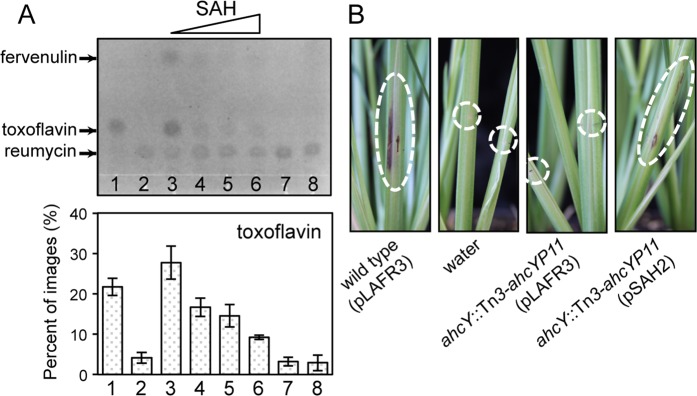
Inhibition of ToxA activity with increasing concentrations of SAH. (**A**) Biosynthesis of toxoflavin *in vitro* (upper panel) and quantitative analysis of toxoflavin biosynthesis (bottom panel). 1, toxoflavin (25 µM); 2, reumycin (25 µM); 3, ToxA (1 µM) plus reumycin (25 µM) plus SAM (125 µM); 4, ToxA (1 µM) plus reumycin (25 µM) plus SAM (125 µM) plus SAH (750 µM); 5, ToxA (1 µM) plus reumycin (25 µM) plus SAM (125 µM) plus SAH (1,000 µM); 6, ToxA (1 µM) plus reumycin (25 µM) plus SAM (125 µM) plus SAH (1,250 µM); 7, ToxA (1 µM) plus reumycin (25 µM); 8, reumycin (25 µM) plus SAM (125 µM). (**B**) Pathogenicity assays of the wild type BGR1, the *ahcY* mutant BAH2, and its complementation strain in the stems of rice plants (*Oryza sativa* cv. Milyang23). Rice stems were photographed 7 days after injection.

To demonstrate that excess amounts of SAH inhibit ToxA activity *in vitro*, we used purified ToxA-His, with reumycin as a precursor and SAM as a methyl donor, for toxoflavin synthesis. ToxA activity was hindered by increasing the molar ratio of SAH to SAM *in vitro* (Fig. 6A). These results confirmed that reumycin is a precursor of toxoflavin and that ToxA is indeed a SAM-dependent methyltransferase. When we evaluated the virulence of BAH2 in rice stems, rice infected with the mutant strain did not show any visible symptoms, whereas both the wild type and the pSAH2-complemented strain of BAH2 caused discoloration in rice stems (Fig. 6B). This indicates that the virulence of the *ahcY* mutant was eliminated due to lack of toxoflavin production, but also due to retarded growth.

## Discussion

While AHL synthases are known to use SAM and acyl-ACP as substrates for the synthesis of AHL QS signals, it remains to be determined how the recycling of these substrates is managed in AHL-mediated QS bacteria. Recycling of SAM requires a continuous source of ATP to add an adenosyl group to methionine and is essential for cellular methylation and biosynthesis of AHL signals as bacterial cell density increases. We found that QS-dependent SAM recycling and metabolic homeostasis of the AMC are both critical to the process by which QS bacteria tune their primary metabolism to cooperative environments.

The AMC is an important metabolic cycle that, in addition to its previously known biochemical processes^17–19^, provides precursors for AHL and AI-2 signal biosynthesis by QS bacteria. QS-dependent control of the AMC appears to be common in other AHL-producing QS bacteria. For example, SAM-dependent methyltransferase genes appear to be controlled by QS in other AHL-producing bacteria, as shown in Table 1. The AHL-mediated activation of *toxA* and the other two SAM-dependent methyltransferase genes (bglu_1g23220 and bglu_2g17510) in *B*. *glumae* clearly shows that QS is involved in cellular methylation processes. These findings are consistent with our previous reports that expression of toxoflavin biosynthetic and transport genes are under the control of QS^15^. In *Photorhabdus luminescens*, transcriptome analysis showed that the expression of SAM-dependent methyltransferase genes might be controlled by AI-2-mediated QS^20^. However, it is not known whether cellular methylation occurs in an AI-2-dependent manner in *P*. *luminescens*.

**Table 1:** Comparison of genes involved in the AMC of AHL-producing bacteria.

Species	*ahcY*	*metF*	*metE*	*metH*	*metK*	QS-dependent SAM-MTases
*Burkholderia glumae* BGR1^a^	**bglu_1g01990**	**bglu_1g02010**	- ***metE1*** **(bglu_1g08430)** - ***metE2*** **(bglu_2g04930)**	- *metH1* (bglu_1g32280) - *metH2* (bglu_1g32290)	bglu_1g33680	- **bglu_2g06400** - **bglu_1g23220** - **bglu_2g17510**
*Burkholderia thailandensis* E264^b^	**BTH_I3165**	**BTH_I3163**	BTH_I1606	- *metH1* (BTH_I0358) - *metH2* (BTH_I0357)	BTH_I0174	- **BTH_II1307** - **BTH_II1338** - **BTH_I2673**
*Burkholderia pseudomallei* 1026b^c^	BP1026B_I3525	BP1026B_I3523	BP1026B_I0767	- *metH1* (BP1026B_I3121) - *metH2* (BP1026B_I3120)	BP1026B_I3302	- **BP1026B_I0156** - **BP1026B_I0642** - **BP1026B_I0822** - **BP1026B_I2850** - **BP1026B_II1162**
*Pseudomonas aeruginosa* PAO1^d^	PA0432*	PA0430*	PA1927*	PA1843*	PA0546	- **PA4209** - **PA0510**

^a^Gene IDs and regulations by QS of *B*. *glumae* BGR1 were obtained from An *et al*.^32^. Bold letters indicate genes activated by QS.

^b^Gene IDs and regulations by QS of *B*. *thailandensis* E264 were obtained from Majerczyk *et al*.^46^. Bold letters indicate genes activated by QS.

^c^Gene IDs and regulations by QS of *B*. *pseudomallei* 1026b were obtained from Majerczyk *et al*.^47^. Bold letters indicate genes activated by QS.

^d^Gene IDs and regulations by QS of the *P*. *aeruginosa* PAO1 were obtained from Schuster *et al*.^48^, Wagner *et al*.^49^, and Asfahl *et al*.^24^. Bold letters indicate genes activated by QS; asterisk denotes derepression in the absence of a QS anti-activator, QteE/QslA.

For *de novo* methionine biosynthesis in *E*. *coli* and *Salmonella typhimurium*, MetJ and SAM are involved in the repression of methionine biosynthetic gene expression as a repressor and a corepressor, respectively^21,22^. Expression of *metF* and *metE* is activated by MetR, the transcriptional activity of which is modulated by homocysteine^23^. The mediation of gene regulation by MetR and MetJ seen in *E*. *coli* was not expected to occur in *B*. *glumae*, due to its lack of MetJ. In *P*. *aeruginosa*, expression of *ahcY*, *metF*, and *metE* is derepressed by the absence of the QS anti-activator, QteE/QslA^24^ (Table 1). However, it is not known whether *ahcY* and *metF* genes are co-transcribed in other bacteria as they are in *B*. *glumae*. Such genetic organization of *ahcY*-*metF* is not commonly found in other QS bacteria^25^. MetR might act a transcriptional activator for *metE*, *metH*, and *metF* expression in *B*. *glumae*, but its role was not determined in this study. It is clear that expression of *metE1* is controlled by QS; however, the nature of simultaneous binding of TofR and QsmR to the putative promoter region of *metE1* remains unclear. In *E*. *coli* and S. *typhimurium*, transcriptome analysis showed that expression of *metE* might be controlled by AI-2-mediated QS^26,27^. However, this requires further confirmation since the majority of *luxS*-responsive genes were not influenced by the exogenous AI-2 in the transcriptome analysis^28,29^.

Unlike *B*. *glumae*, many other bacteria do not carry both *metE* and *metH* for the biosynthesis of methionine from homocysteine^30^. Since *P*. *aeruginosa* is devoid of cobalamin biosynthetic genes, it is conceivable that it has vitamin B_12_-dependent MetH for successful interaction with its hosts. However, *B*. *glumae* carries active cobalamin biosynthetic genes^31^ and does not require vitamin B_12_ for MetH activity. It is not currently understood why *B*. *glumae* possesses redundant *met* genes.

Since SAM is used as a substrate of AHL QS signal synthases, it is important to understand how the cellular availability of SAM fluctuates as cell density increases. Higher cellular concentrations of SAM in two QS mutants, BGS2 and BGS9, than in the wild type were predictable because of null mutations in *tofI* in the mutant strains. However, quantification of methionine concentrations does not always reflect the level of *de novo* biosynthesis. One plausible explanation for the higher methionine concentrations seen in BGS2 and BGS9 than in the wild type is a phenomenon of QS-mediated metabolic slowing in *B*. *glumae*^32^. Cellular concentrations of homocysteine were not influenced by AHL-mediated QS and remained relatively stable, which might be due to an alternative pathway for homocysteine biosynthesis from homoserine^30,33^.

Conversion of SAH into homocysteine by SAH hydrolase and Pfs/LuxS removes the cellular toxicity of accumulated SAH in cells^7–9^. Thus, biosynthesis of AI-2 mediated by LuxS in a variety of gram-negative and gram-positive bacteria is not only the process by which a bacterial communication signal is produced but also has metabolic roles. For instance, mutations in *luxS* and *pfs* genes caused metabolic perturbations in AMC intermediates in *E*. *coli*^34^. Inactivation of *pfs* or *luxS* in AI-2-producing pathogenic bacteria impaired virulence via negative effects on exoenzyme and toxin production, motility, and biofilm formation^20,35–38^. However, considering that LuxS is involved in the AMC, it is not clear whether the phenotypic differences between the *luxS* mutant and its parent strain are the result of the lack of an AI-2 signal, metabolic perturbation, or both.

As observed in *B*. *glumae*, the negative impact of excess SAH on SAM-dependent methyltransferase activity has been shown in *Rhodopseudomonas spheroids*, a species in which a SAM-dependent methyltransferase plays a critical role in bacteriochlorophyll biosynthesis^39,40^. As a competitive inhibitor of SAM, SAH also inhibits AHL synthase activity *in vitro*^41^. In the non-polar *ahcY* mutant BAH2, ATP was present in significantly lower quantities than in the wild type, contributing to the imbalance between SAM and SAH. We speculate this might be another phenomenon caused by the negative influences of the imbalance between SAM and SAH. The ATP synthase c-subunit is methylated by a SAM-dependent methyltransferase, but lack of methylation has been shown to hinder the intact assembly of the ATP synthase complex, thereby reducing ATP production, in human mitochondria^42^. The imbalance of SAM and SAH and low level of ATP in the *ahcY* mutant BAH2 explains its relatively slow growth compared to the wild type, even though slow growth of the mutant is probably due to accumulation of toxic SAH. In *E*. *coli*, *Streptococcus pyogenes*, and *Neisseria meningitides*, it has been speculated that growth defects of the AI-2 mutants might be due to toxic effects of accumulated SAH^34,43,44^.

**Figure 7:**
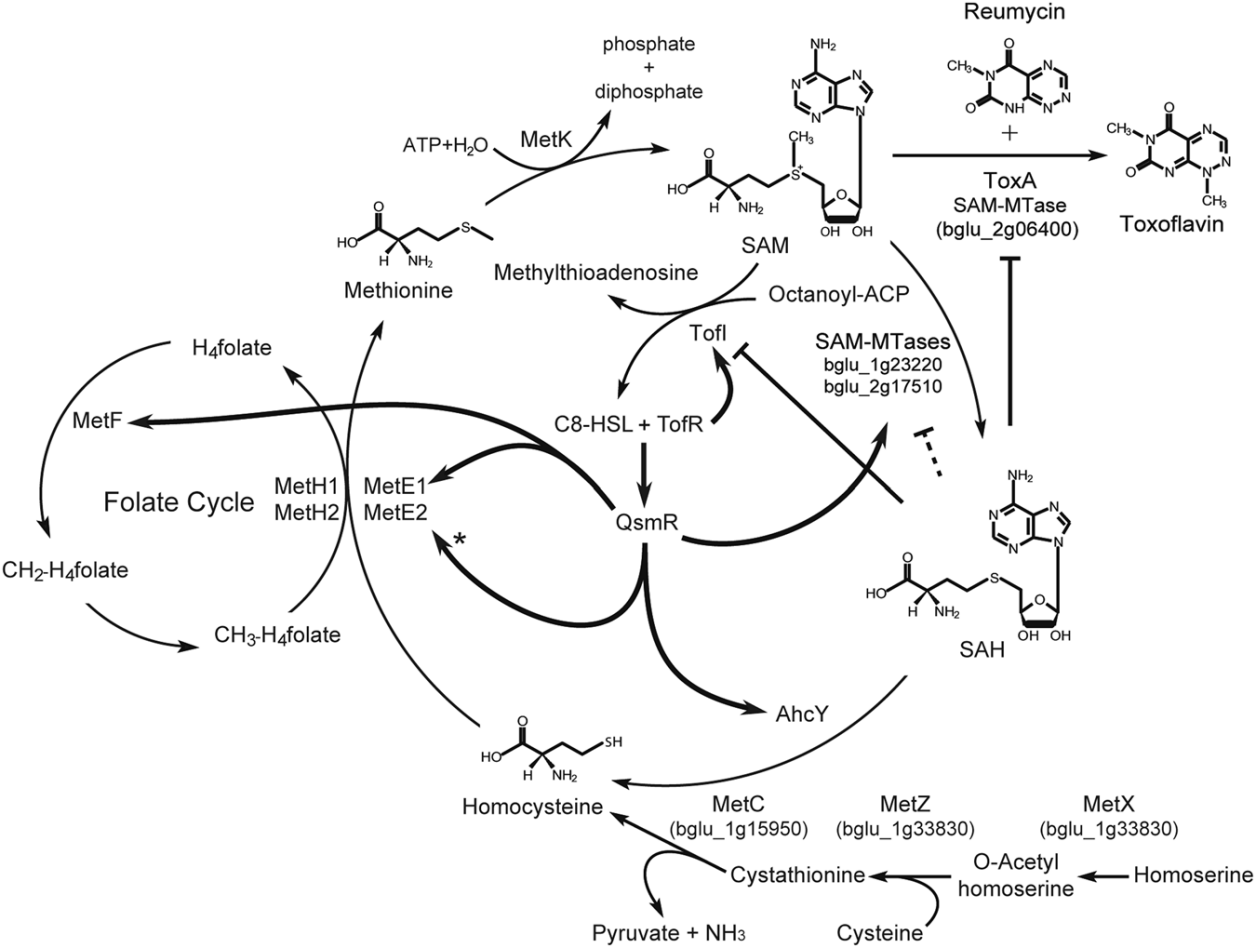
Regulation of genes involved in the AMC by QS in *B*. *glumae*. SAH, S-adenosyl-L-homocysteine; SAM, *S*-adenosyl-L-methionine; AhcY, SAH hydrolase (gene ID: bglu_1g01990); MetK, SAM synthase (gene ID: bglu_1g33680); MetF, methylenetetrahydrofolate reductase (gene ID: bglu_1g02010); MetE1, 5-methyltetrahydropteroyltriglutamate-homocysteine methyltransferase 1 (gene ID: bglu_1g08430); MetE2, 5-methyltetrahydropteroyltriglutamate-homocysteine methyltransferase 2 (gene ID: bglu_2g04930); MetH1, methionine synthase 1 (gene ID: bglu_1g32280); MetH2, methionine synthase 2 (gene ID: bglu_1g32290); octanoyl-ACP, octanoyl acyl-carrier protein; C8-HSL, *N*-octanoyl homoserine lactone; MetX, homoserine O-acetyltransferase (gene ID: bglu_1g33830); MetZ, O-acetylhomoserine sulfhydrylase (gene ID: bglu_2g08500); MetC, cystathionine beta-lyase (gene ID: bglu_1g15950). T shape indicates inhibition; dashed T indicates putative inhibition. Bold arrows indicate activation, and the asterisk denotes QsmR-dependent expression of *metE2* in the absence of MetH1/H2.

These results demonstrate that, along with other various gene regulation networks controlled by bacterial QS, control of the AMC by AHL signals is a typical positive feedback control circuit critical for sustainable production of AHL signals to ensure accurate representation of population density in cooperative populations (Fig. 7). In addition to QS-mediated metabolic slowing^32^, metabolic homeostasis of the AMC, and especially of the ratio between SAM and SAH, is crucial to the synthesis of AHL signals to reflect changes in cell density. These results illustrate another critical connection between bacterial primary metabolism and cell density-dependent gene regulation systems.

## Methods

### Bacterial strains and growth

The bacterial strains and plasmids used in this work are shown in Supplementary Table 3. Strains of *B*. *glumae* were grown at 37 °C in LB medium [1% (w/v) tryptone and 0.5% (w/v) of yeast extract (pH 7.0); USB Corp., Cleveland, OH, USA] or M9 minimal medium (Na_2_HPO_4_, KH_2_PO_4_, NaCl, and NH_4_Cl) with glucose (0.2%) added as a carbon source. Antibiotics were used at the following concentrations: ampicillin, 100 µg mL^−1^; chloramphenicol, 20 µg mL^−1^; kanamycin, 50 µg mL^−1^; nalidixic acid, 20 µg mL^−1^; rifampicin, 100 µg mL^−1^; spectinomycin, 100 µg mL^−1^; tetracycline, 10 µg mL^−1^; and gentamicin, 20 µg mL^−1^.

### DNA construction and mutagenesis

General and standard techniques were used for DNA manipulations, cloning, restriction digestions, and agarose gel electrophoresis^45^. The genetic information and gene IDs for DNA construction were obtained from the *B*. *glumae* BGR1 genome database (GenBank accession numbers: CP001503–CP001508). Mutagenesis of genes with Tn*3-gusA* was performed as described previously^15^. Mutagenized plasmids were introduced individually by conjugation followed by marker-exchange into the *B*. *glumae* strains as described previously^15^. A 10.0-kb KpnI DNA fragment harboring *ahcY* was cloned into pLAFR6, resulting in pSAH4 (Fig. 4). A 3.7-kb SacII DNA fragment harboring a putative promoter region, *ahcY*, *pmp1*, and *metF* from pSAH4 was first cloned into pBluescript II SK(+) followed by recloning into pLAFR3 using the unique BamHI and SacI sites to yield pSAH5 (Supplementary Table 3). To construct an *ahcY* mutant, the 310-bp region upstream of *ahcY* was amplified by PCR using specific primers (Supplementary Table 2). The amplified DNA fragment carrying a putative *ahcY* promoter region was replaced into *gusA* using the unique EcoRI site. The resulting plasmid, pSAH4::Tn*3-ahcYP11* was introduced by conjugation and marker-exchanged into the wild type strain BGR1 (Fig. 4). The gentamicin cassette was inserted into the EcoRI or BamHI site in *metE2* or *metR2* in pMETH2. Similarly, the Ω cassette was inserted into the unique BamHI site in the *metH1* and *metH2* sequence in pMETH (Supplementary Fig. 2). These plasmids were introduced into *B*. *glumae* strains to generate the *metE2*-*metR2*::Gm^r^ and *metH1-2*::Ω mutant strains, respectively, via marker-exchange mutagenesis. To obtain *B*. *glumae metH* and QS double mutants, we used cosmid clones pBGA18 and pBGF6 containing *tofI* (bglu_2g14490) and *qsmR* (bglu_1g10250), respectively (Supplementary Table 3), and subjected *B*. *glumae* to mutagenesis using EZ-Tn5^TM^ < DHFR-1 > (Epicentre, Madison, WI, USA), as described in the manufacturer’s protocols. Each single Tn5 insertion in *tofI* or *qsmR* was marker-exchanged into *B*. *glumae* BMH1 (BGR1 *metH1-2*::Ω), resulting in BMH2 and BMH9, respectively. Each Tn5 insertion in *tofI* or *qsmR* was marker-exchanged into *B*. *glumae* BMEH1 (BGR1 *metE1*::Tn*3-gusA36*/*metH1-2*::Ω) to obtain *metE*, *metH*, and the QS triple mutants BMEH2 and BMEH9. All marker-exchanges were confirmed by Southern hybridization analysis using each cosmid as a probe. For *ahcY* complementation, we deleted a 17.9-kb BamHI DNA fragment from pSAH1::Tn*3-gusA26* as shown in Fig. 4. The resulting plasmid pSAH2 was introduced into BAH2 (*ahcY*::Tn*3-ahcYP11*) by conjugation.

### Reverse transcription (RT)-PCR and quantitative real-time (qRT)-PCR analysis

The *B*. *glumae* strains were inoculated with an optical density at 600 nm (OD_600_) of 0.05) and grown in 2 mL of LB medium for 10 h at 37 °C to reach the late exponential phase. Total RNA extraction was performed using a RNeasy Mini Kit (Qiagen, Venlo, Netherlands) according to the manufacturer’s instructions. Isolated RNA was treated with DNaseI (Qiagen) for 30 min at 37 °C. To remove genomic DNA completely, isolated RNA was treated with DNaseI (Ambion, Austin, TX, USA) for 1 h at 37 °C according to the supplier’s instructions. A total of 1 µg RNA was reverse transcribed into cDNA using M-MLV reverse transcriptase (Promega, Madison, WI, USA) for 1 h at 42 °C. PCR was performed using a PTC-200 Thermo Cycler (MJ Research, Waltham, MA, USA) with the following conditions: 96 °C for 5 min, followed by 25 cycles of 96 °C for 1 min, 55 °C for 30 s, and 72 °C for 2 min. The primers used for each RT-PCR and qRT-PCR reactions are listed in Supplementary Table 2. For the quantification of transcriptional levels, qRT-PCR was performed using SsoFast EvaGreen Supermix (Bio-Rad, Hercules, CA, USA) on a C1000 Thermal Cycler CFX96 Real-Time PCR Detection System (Bio-Rad). The thermal cycling parameters were 95 °C for 2 min, followed by 40 cycles of 98 °C for 2 s, 55 °C for 5 s, and 72 °C for 10 s. PCRs were repeated three times, and the fold change in the target genes was normalized to 16S rRNA reference genes using CFX Manager Software (Bio-Rad). The PCR machine shows the PCR cycle threshold (*Ct*) value of each sample and analyzes results automatically.

### Overexpression and purification of QsmR, TofR, and ToxA

QsmR-His and His-TofR were overexpressed and purified as described previously^16^. To overexpress ToxA in *E*. *coli*, the coding region of *toxA* was amplified using pBGT7 as the template DNA and the oligonucleotide primers ToxA1-F (5′-CCGTTGATATTGAAAGGTTACATATGAGTAC-3′) and ToxA1-R (5′-ATCGAACCGCCCCTGGCAAGCTTAG-3′), which introduced unique NdeI and HindIII sites at the ends of the PCR product. The amplified product was cloned into the corresponding sites of pET21b (Invitrogen, Eugene, OR, USA), resulting in pToxA-His (Supplementary Table 3). ToxA-His was overexpressed in *E*. *coli* strain BL21(DE3), as described by the manufacturer (Novagen, Hornsby Westfield, Australia). Soluble ToxA-His was purified in a buffer containing 20 mM Tris-HCl (pH 7.7), 0.1 mM EDTA, 1 mM dithiothreitol, 5% glycerol, and 200 mM NaCl, using an Ni-NTA spin column as described by the manufacturer (Qiagen).

### Gel mobility shift assay

The 326-bp region upstream of *ahcY* and the 181-bp region upstream of *metE1* were amplified by PCR using specific primers (Supplementary Table 2). The fragment was labeled with biotin for chemiluminescence using a LightShift Chemiluminescent Electrophoretic Mobility Shift Assay Kit (Thermo Fisher Scientific Inc., Waltham, MA, USA). For competitor DNA, we used the 260-bp upstream region of *lexA* and the 242-bp upstream region of *katE* were amplified by PCR using LexA-F and LexA-R primers and KEN1 and KEN2 primers respectively (Supplementary Table 2). Purified QsmR-His and His-TofR were incubated with 0.4 nM biotin-labeled DNA in a binding buffer [10 mM Tris-HCl (pH 7.5), 10 mM NaCl, 100 mM NaCl, 0.5 mM EDTA, 1 mM dithiothreitol, 5% (v/v) glycerol, and 50 ng µL^−1^ poly(dI·dC)] for 15 min at 28 °C. A 32-fold molar excess of unlabeled target DNA was added to the reaction along with the extract, before the addition of the labeled DNA target. The mixtures were size-fractionated on a non-denaturing 4% polyacrylamide gel followed by drying and transfer to nitrocellulose membranes. Signals were detected as described by the manufacturer (Thermo Fisher Scientific Inc.).

### Measurement of the cellular concentrations of methionine, SAM, SAH, homocysteine, and adenine triphosphate (ATP)

Cellular metabolites of the wild type BGR1, BGS2 (BGR1 *tofI*::Ω), BGS9 (BGR1 *qsmR*::Ω), BAH2 (BGR1 *ahcY*::Tn*3-ahcYP11*), and complementation strain BAH2 (BGR1 *ahcY*::Tn*3-ahcYP11*/pSAH2) were extracted as follows. Aliquots (2 mL) were collected 10 h after subculture and normalized by cell counting. Cells were washed with 20 mL of Milli-Q water to remove any residual medium and lysed by sonication with a VCX-400 sonicator (Sonics & Materials, Newton, CT, USA). The lysates were cleared by centrifugation at 10,000 × *g* for 20 min. To measure cellular methionine and ATP concentrations, the lysates were analyzed using a Dionex Ultimate 3000 high-performance liquid chromatography system (Thermo Dionex, Waltham, MA, USA) equipped with an Agilent 1260 Infinity FL Detector (Agilent, Santa Clara, CA, USA). Liquid chromatography separation was performed using an Inno C18 column (4.6 mm × 150 mm, 5 µm; Youngjin Biochrom, Seongnam, Korea) with a mobile phase A (40 mM sodium phosphate, pH 7) and a mobile phase B [water/acetonitrile/methanol (10:45:45 v/v)]. The flow rate was 0.25 mL min^−1^ and the injection volume was 0.5 μL. The levels of homocysteine, SAM, and SAH in *B*. *glumae* strains were measured using ELISA kits for homocysteine, SAM, and SAH (Cell Biolabs, San Diego, CA, USA) according to the manufacturer’s instructions.

### Toxoflavin and C8‐HSL production assays

The toxoflavin and autoinducer production assays were performed as described previously^15^.

### *In vitro* biosynthesis of toxoflavin and its inhibition by SAH

SAM and SAH were purchased from Sigma Aldrich Co Ltd (St. Louis, MO, USA). Reumycin and toxoflavin were provided by Tomohisa Nagamatsu. For *in vitro* biosynthesis of toxoflavin, ToxA-His was purified as described above, and mixed with SAM and reumycin in 400 µL of a solution of 0.1 M sodium phosphate buffer (pH 7.0). Reaction mixtures were incubated for 1 min at 37 °C. For inhibition of toxoflavin biosynthesis by SAH, ToxA-His was mixed with SAM, reumycin, and various concentrations of SAH in 400 µL of a solution of 0.1 M sodium phosphate buffer (pH 7.0). The extraction of samples was performed as described for the toxoflavin assays. After development of TLC, the images were visualized at 312 nm using Chemi Doc XRS+ with Image Lab Software (Bio-Rad, Hercules, CA, USA). The amounts of toxoflavin synthesized were quantified using standards of known concentrations of toxoflavin, as described previously^15^.

### Immunoblotting using an anti-*S*-adenosylmethionine (SAM) synthase antibody

Cellular proteins from *B*. *glumae* strains were separated by 12% sodium dodecyl sulfate polyacrylamide gel electrophoresis and transferred to a polyvinylidene fluoride membrane. The blocked membrane was incubated with a rabbit polyclonal antibody against the N-terminal region of SAM synthase (MetK; NBP1-55120; Novus Biologicals, LLC, Centennial, CO, USA). The membrane was then probed with a rabbit IgG-HRP antibody (Life Technologies, Carlsbad, CA, USA), which was followed by visualization with a chemiluminescent substrate (Bio-Rad).

### Statistical analysis

All statistical analyses and ANOVA testing followed by Tukey’s honest significance difference post hoc analysis were performed using IBM SPSS Statistics software (ver. 20x86-x64; IBM Corp., Armonk, NY, USA).

## Supplementary information

Supplementary Information
