# Supplementary material for: Unraveling the role of quorum sensing-dependent metabolic homeostasis of the activated methyl cycle in a cooperative population of Burkholderia glumae
Source: Sci Rep. 2019 Jul 30;9:11038. doi: 10.1038/s41598-019-47460-6 (PMC6667456; doi:10.1038/s41598-019-47460-6)
Supplement: Supplementary file 1 — Supplementary Information [file 41598_2019_47460_MOESM1_ESM.pdf]

Supplementary information for

Unraveling the role of quorum sensing-dependent metabolic homeostasis of the activated methyl cycle in a cooperative population of *Burkholderia glumae*

Yongsung Kang, Hongsup Kim, Eunhye Goo, Hyesung Jeong, Jae Hyung An, and Ingyu Hwang

Ingyu Hwang
ingyu@snu.ac.kr

Supplementary Table 1 RNA sequencing analysis of SAM-dependent methyltransferases in *B. glumae*

Gene ID[a]	Gene	Reads per kilobase per million mapped reads (RPKM)					
		BGR1 6 h	BGS2 6 h	BGS9 6 h	BGR1 10 h	BGS2 10 h	BGS9 10 h
bglu_1g23220		116.297	25.337	49.856	140.478	10.317	45.128
bglu_2g17510		191.975	46.455	161.264	833.088	423.280	276.659
bglu_2g06400	*toxA*	10956.921	2512.899	9521.271	7943.327	12675.912	6800.877

[a] gene IDs were obtained from the *B. glumae* BGR1 genome database (GenBank accession numbers: CP001503–CP001508).

Supplementary Table 2 Primers used for PCR and qRT-PCR

Primer name	Sequence (5′→ 3′)
1g23220-F	GAATTTCAGCGTGGACACATT
1g23220-R	CAGATGGCCTTCATGTTTCA
2g17510-F	CAGGTATTCCGCGTGCTG

2g17510-R	CCCAGTGGGTATCGATGATT
PCR1-F	CCCGGCCTTCAACGTCAACG
PCR1-R	ACCACGCGGTAGCCTTCCAT
PCR1-C	CGGCCGCGTATTCCATGGTC
PCR2-F	ATCATCAATGCCCTCGCGCT
PCR2-R	TCGAAGCCCTTCAGCAGCGA
PCR2-C	CCGAAGAACGCCGACCAGAA
PCR3-F	ATCGGCGCCTCGAAGGACAA
PCR3-R	TCGGCGAAGTGCAGCAGGTC
PCR3-C	AAGTAGGCGTCCGCGTTGAA
16S rRNA-F	TCTGAGAGGACGACCAGCCA
16S rRNA-R	CGAAGGCCTTCTTCACACAC
AhcY-F	AGCCGAACACTGAGAGGACG
AhcY-R	CATCACGCCCTCCTTTCTAATC
AhcY1-F	GAGGACGCGCGTAGAGGACG
AhcY1-R	GTTCATCACGCCCTCCTTTCT
ToxA1-F	CCGTTGATATTGAAAGGTTACATATGAGTAC
ToxA1-R	ATCGAACCGCCCCTGGCAAGCTTAG
ToxA-F	ATGATCGAGCTGGCCCGTGA
ToxA-R	CGGGTCGACGGTATAGGCCA
ToxA-C	TTGAGCACGTTCACGCCGTA
TofI-F	TGGGGAGATATGCGGTTGCG
TofI-R	CGTGACCCCGATCAACTGGC
TofI-C	AGCCGCTCGATGCTGCAGAA
MetF-C	TAGAAGTGCAGCCCCGGCGC
HpoT-F	CTTCGAGGTGTCCGGCTTCT
HpoT-R	CCAGGGAGAGTTCGATCGGT
SahH-F	AGCAGGCGTCGTACATCGGC
SahH-R	GGTGATGATGAGCAGCGCGA
MetE1-F	GAGAACTGGCGGATGCAGCG

MetE1-R	ATTCCGGCACCACGTAACGA
MetE1-C	GCCTCCGCCACCTCGTCGAA
MetE1PF	AGCGCCGTGAGGGTCTGCAG
MetE1PR	TGGTGTCCTCCAGATATGGGCT
KEN1	ATCGGCCAAACGCAGCA
KEN2	AACGGCGGCATCAGCAA
MetE2-F	TCGAGCGCTACTGGAAGGGC
MetE2-R	CGGAAGCTGTTGTCGAGCGC
MetE2-C	CCACTTCGTCATCTCGCCGG
MetK-F	GACAAATACTCGCGTGTTGC
MetK-R	CCTTGTAGTCGATGCCGAAG
MetK-C	ATTCTCGATGCCATCCTCAC

Supplementary Table 3 Strains and plasmids used in this study

Bacterial strain or plasmid	Genotype or phenotype[a]	Reference or source
Escherichia coli		
DH5α	F$^-$Φ80d*lac*ZΔ*M15*Δ(*lac*ZYA-*argF*)*U169endA1 recA1hsd1hsdR17*($r_k^-m_k^+$)*deoRthi*-1*supE44*λ$^-$ *gyrA96 relA1*	Gibco BRL
C2110	*polA*, Nal^r	1
HB101	F$^-$*mcrBmrrhsdS20*($r_B^-m_B^-$)*recA13leuB6ara-14 proA2lacY1galK2xyl-5 mtl-1 rpsL20*(Sm^r) *supE44*λ$^-$	Gibco BRL
BL21(DE3)	F-*ompT hsdS$_B$*($r_B^-m_B^-$)*gal dcm* (DE3)	Novagen
JW3805	Δ(*araD-araB*)567 Δ*lacZ*4787(::*rrnB*-3) λ$^-$rph^{-1} Δ*metE*774::kan Δ(*rhaD-rhaB*)568 *hsdR*514	2
Agrobacterium tumefaciens		
NT1(pDCI41E33)	Autoinducer indicator strain	3

Burkholderia glumae		
BGR1	Wild type, Rif^{r}	4
BGS2	BGR1 *tofI*::Ω	4
BGS9	BGR1 *qsmR*::Ω	5
BAH1	BGR1 *ahcY*::Ω	This study
BAH2	BGR1 *ahcY*::Tn*3-ahcYP11*	This study
BPM1	BGR1 *pmp1*::Tn*3-gusA197*	This study
BMF1	BGR1 *metF*::Tn*3-gusA43*	This study
BME1	BGR1 *metE1*::Tn*3-gusA36*	This study
B2ME1	BGS2 *metE1*::Tn*3-gusA36*	This study
B9ME1	BGS9 *metE1*::Tn*3-gusA36*	This study
BMH1	BGR1 *metH1-2*::Ω	This study
BMEH1	BGR1 *metE1*::Tn*3-gusA36*/*metH1-2*::Ω	This study
BME12	BGR1 *metE1*::Tn*3-gusA36*/*metE2-metR2*::Gm^{r}	This study
BMH2	BGR1 *tofI*::Tn*5*/*metH1-2*::Ω	This study
BMH9	BGR1 *qsmR*::Tn*5*/*metH1-2*::Ω	This study
BMEH2	BGR1 *tofI*::Tn*5*/*metH1-2*::Ω/*metE1*::Tn*3-gusA36*	This study
BMEH9	BGR1 *qsmR*::Tn*5*/*metH1-2*::Ω/*metE1*::Tn*3-gusA36*	This study
Plasmids		
pRK2013	Tra^{+}, ColE1 replicon, Km^{r}	6
pHoKmGus	Promoterless β-glucuronidase gene, Km^{r}, Amp^{r}, *tnpA*	7
pSShe	Cm^{r}	1
pBluescript II SK(+)	Cloning vehicle; phagemid, pUC derivative, Amp^{r}	Stratagene
pLAFR3	Tra^{-}, Mob^{+} RK2 replicon, Tet^{r}	8
pLAFR6	As pLAFR3 but without *lacZα*, contains multilinker of pUC18 flanked by synthetic *trp*	9

	terminators, Tet^{r}	
pRK415	Mob^{+}, *lacZ*, Tet^{r}	10
pET21b	T7 promoter-based expression vector, Amp^{r}	Novagen
pToxA-His	*toxA* in pET21b, Amp^{r}	This study
pBGT7	28.4 kb DNA fragment harboring *toxA* from BGR1 cloned into pLAFR3	4
pBGA18	25.3 kb DNA fragment harboring *tofI* from BGR1 cloned into pLAFR3	4
pBGF6	18.9 kb DNA fragment harboring *qsmR* from BGR1 cloned into pLAFR3	5
pSAH1	21.2 kb DNA fragment harboring *ahcY* from BGR1 cloned into pLAFR3	This study
pSAH2	3.3 kb DNA fragment harboring putative promoter and *ahcY* from pSAH1::Tn*3-gusA26* by deletion of 17.9-kb BamHI fragment	This study
pSAH4	10.0-kb KpnI DNA fragment harboring *ahcY* from BGR1 cloned into pLAFR6	This study
pSAH5	3.7-kb SacII DNA fragment harboring putative promoter region, *ahcY, pmp1* and *metF* from pSAH4 in pLAFR3	This study
pMETH1	37.6 kb DNA fragment harboring *metE1* from BGR1 cloned into pLAFR3	This study
pMETH2	22.2 kb DNA fragment harboring *metE2* from BGR1 cloned into pLAFR3	This study
pMETH	19.5 kb DNA fragment harboring *metH1/metH2* from BGR1 cloned into pLAFR3	This study

[a] Amp^{r}, ampicillin resistance; Cm^{r}, chloramphenicol resistance; Km^{r}, kanamycin resistance; Nal^{r}, nalidixic acid resistance; Rif^{r}, rifampicin resistance; Sm^{r}, streptomycin resistance; Sp^{r}, spectinomycin resistance; Tet^{r}, tetracycline resistance; Gm^{r}, gentamicin resistance.

References

1. Stachel, S. E., An, G., Flores, C. & Nester, E. W. A Tn*3-lacZ* transposon for the random generation of β-galactosidase gene fusion: application to the analysis of gene expression in *Agrobacterium*. EMBO J **4**, 891–898 (1985).

2. Baba, T. et al. Construction of *Escherichia coli* K-12 in-frame, single-gene knockout mutants: the Keio collection. Mol Syst Biol **2**, 2006. 0008; 10.1038/msb4100050 (2006).

3. Cook, D. M., Li, P-L., Ruchaud, F., Padden, S. & Farrand, S. K. Ti plasmid conjugation is independent of vir: reconstitution of the tra functions from pTiC58 as a binary system. J Bacteriol **180**, 1291–1297 (1997).

4. Kim, J. et al. Quorum sensing and the LysR-type transcriptional activator ToxR regulate toxoflavin biosynthesis and transport in *Burkholderia glumae*. Mol Microbiol **54**, 921–934 (2004).

5. Kim, J. et al. Regulation of polar flagellum genes is mediated by quorum sensing and FlhDC in *Burkholderia glumae*. Mol Microbiol **64**, 165–179 (2007).

6. Figurski, D. H. & Helinski, D. R. Replication of an origin-containing derivative of plasmid RK2 dependent on a plasmid function provided in *trans*. Proc Natl Acad Sci USA **76**, 1648–1652 (1979).

7. Bonas, U., Stall, R. E. & Staskawicz, B. J. Genetic and structural characterization of the avirulence gene *avrBs3* from *Xanthomonas campestris* pv. *vesicatoria*. Mol Gen Genet **218**, 127–136 (1989).

8. Staskawicz, B., Dahlbeck, D., Keen, N. & Napoli, C. Molecular characterization of cloned avirulence genes from race 0 and race 1 of *Pseudomonas syringae* pv. *syringae*. J Bacteriol **169**, 5789–5794 (1987).

9. Huynh, T. V., Dahlbeck, D. & Staskawicz, B. J. Bacterial blight of soybean: regulation of a pathogen gene determining host cultivar specificity. Science **245**, 1374–1377 (1989).

10. Keen, N. T., Tamaki, S., Kobayashi, D. & Trollinger, D. Improved broad-host-range plasmid for DNA cloning in gram negative bacteria. Gene **70**, 191–197 (1988).

Supplementary Figure 1

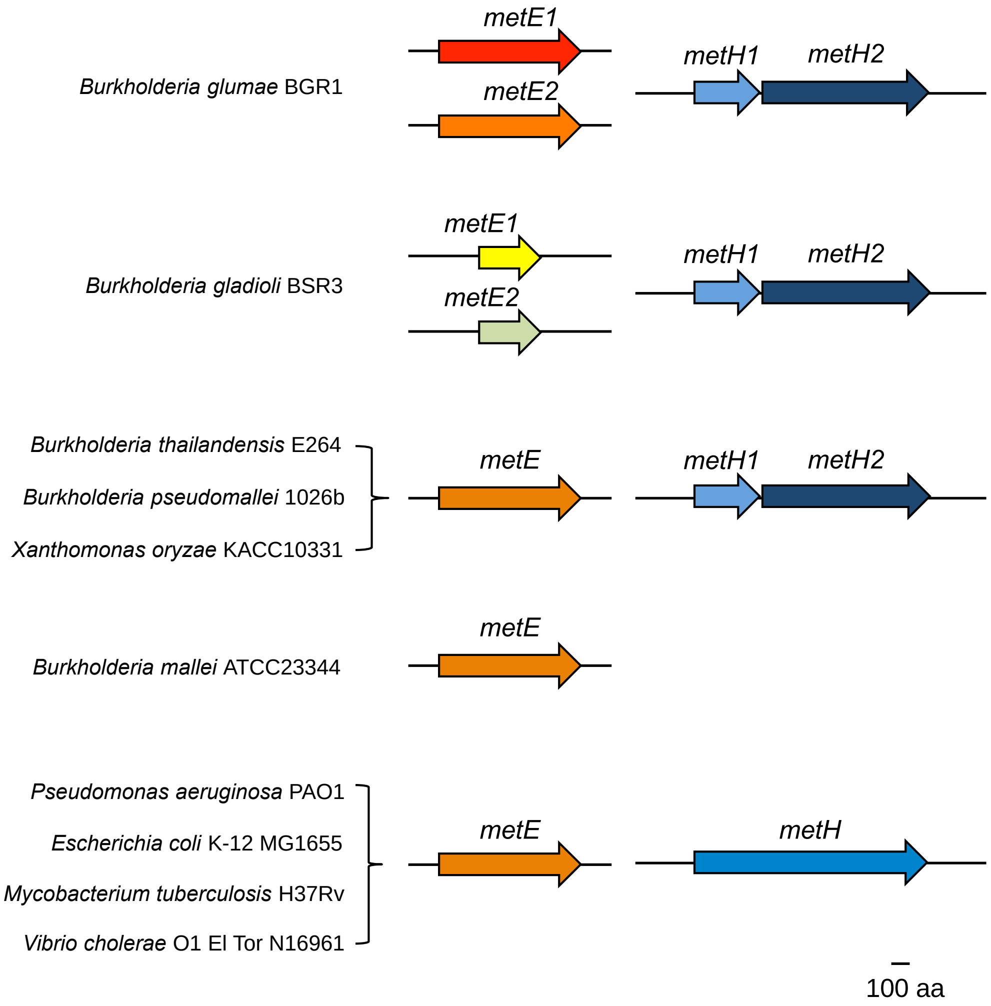

Genetic organization of *metE*/*metH* genes in different bacteria.

Supplementary Figure 2

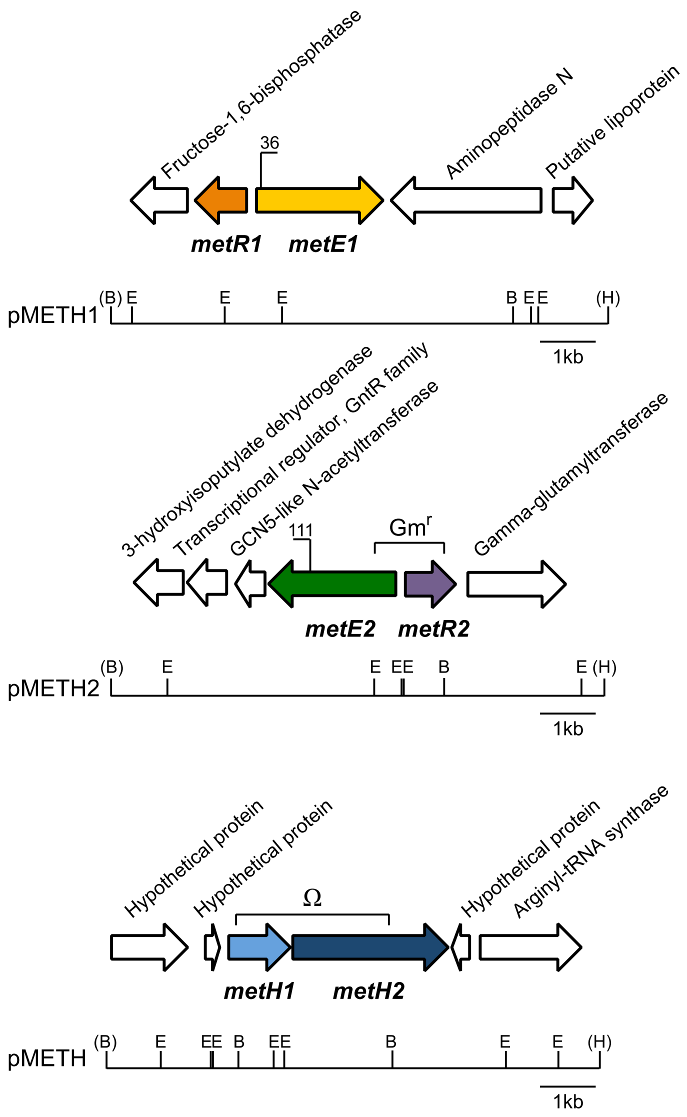

Genetic organization of *metE1*, *metE2*, and *metH1/metH2* genes in *B. glumae* BGR1. The vertical bars in the map denote the positions and orientations of Tn*3-gusA* insertions. The square bracket above the *metE2/metR2* genes indicates the position of gentamicin resistance gene insertion into the EcoRI and BamHI site in pMETH2. The square bracket above the *metH1/metH2* genes indicates insertion of Ω cassette into BamHI site in pMETH. The restriction enzyme sites are indicated as follows: E, EcoRI; B, BamHI; H, HindIII. Enzyme sites from the vector are shown in parentheses.

Supplementary Figure 3

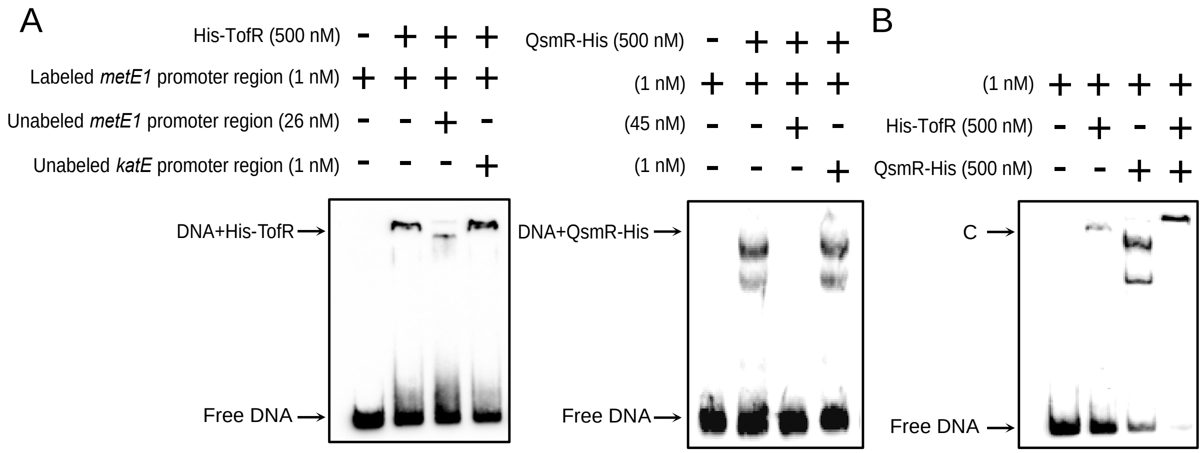

TofR and QsmR directly activate *metE1* expression. (**A**, **B**) Gel mobility shift assays using purified TofR-His and/or QsmR-His to the putative promoter region of *metE1.*

Supplementary Figure 4

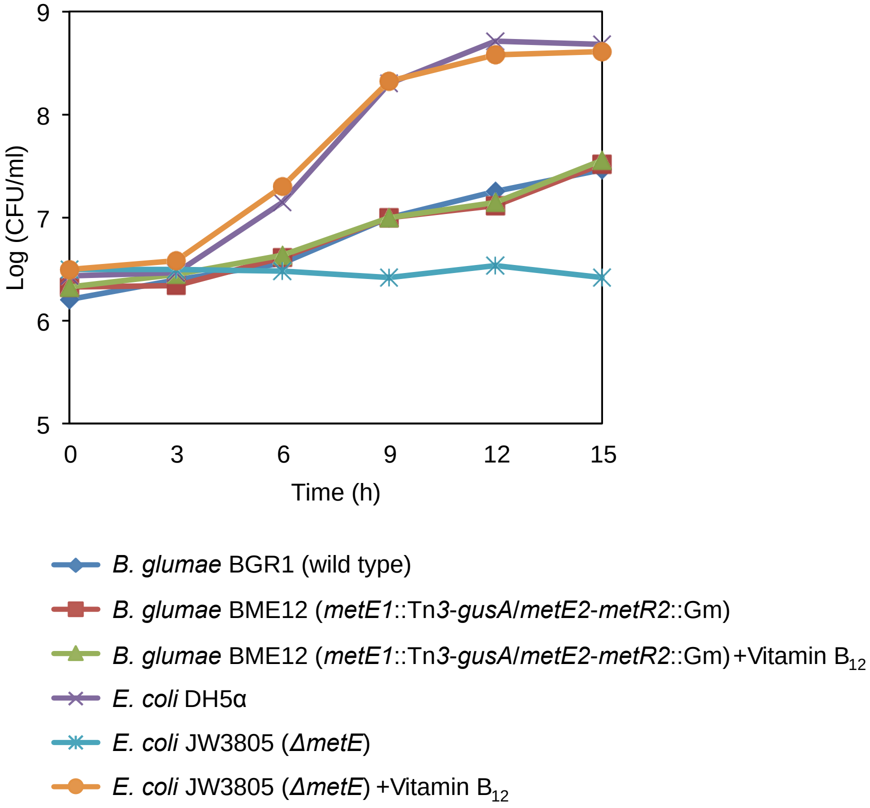

Growth of *metE* mutants of *B. glumae* and *E. coli* in M9 minimal medium. Fresh LB medium was inoculated with overnight culture of *B. glumae* BME12 (BGR1 *metE1*::Tn*3*-*gusA36*/*metE2*-*metR2*::Gm^r), *E. coli* DH5α, or *E. coli* JW3805 (Δ*metE*::Km^r). In the exponential phase, the pre-culture was diluted and grown to an OD_{600} of 0.05 and inoculated into M9 minimal medium containing 0.2% glucose. The growth of *B. glumae* BME12 was independent of vitamin B_{12}, while growth of *E. coli* JW3805 increased to the level of wild-type *E. coli* with the addition of vitamin B_{12}. 5 µM of vitamin B_{12} was added to cultures grown in M9 minimal medium.

Supplementary Figure 5

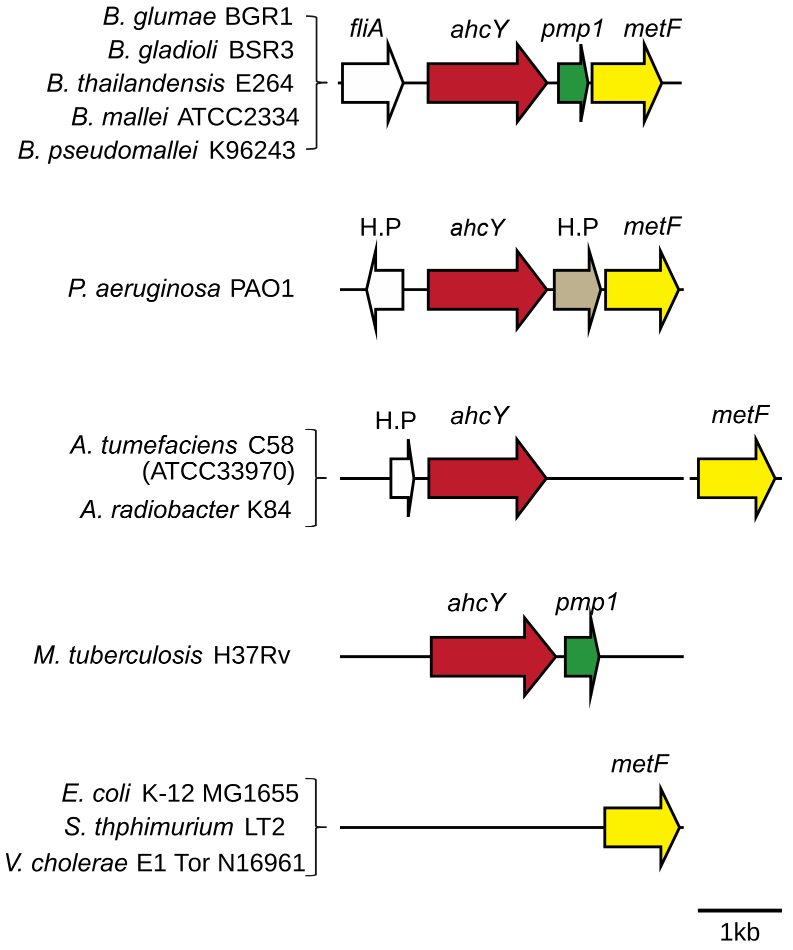

Genetic organization of *ahcY*/*metF* genes in different bacteria. H.P.: hypothetical protein.

Supplementary Figure 6

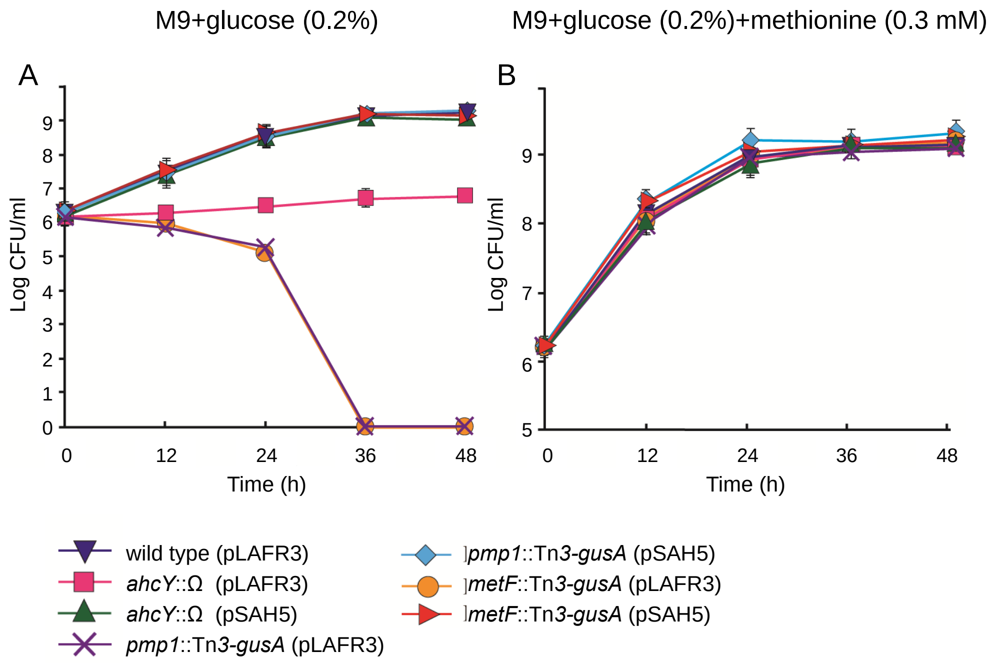

Growth of *ahcY*, *pmp1*, and *metF* mutants was dependent on methionine in M9 minimal media. The following strains were used in this experiment: BGR1 (wild type), BAH1 (BGR1 *ahcY*::Ω), BPM1 (BGR1 *pmp1*::Tn*3-gusA197*), and BMF1 (BGR1 *metF*::Tn*3-gusA43*). (**A**) M9 + glucose (0.2%) minimal medium. (**B**) M9 + glucose (0.2%) + methionine (0.3 mM) minimal medium. All experiments were performed with at least three replicates.

Supplementary Figure 7

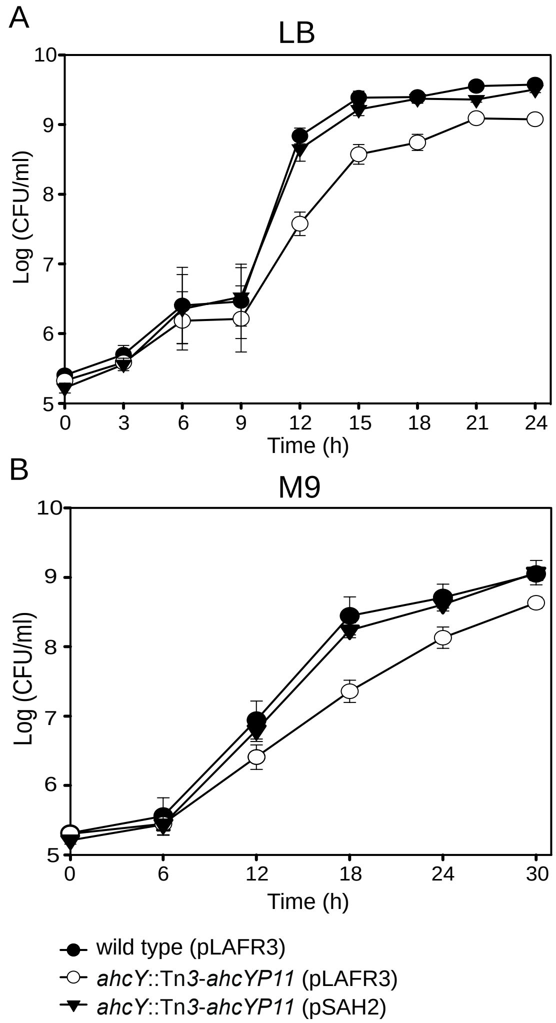

Growth of BAH2 in LB and M9 minimal media. (**A**) Growth of BAH2 in LB medium. (**B**) Growth of BAH2 in M9 minimal medium. The strains used in this experiment were BGR1 (wild type) and BAH2 (BGR1 *ahcY*::Tn*3-ahcYP11*). Fresh LB medium was inoculated with overnight culture of one of the two strains. In the exponential phase, the pre-cultures were diluted and grown to an OD_{600} of 0.05, and then split into two cultures, one of which was inoculated into M9 minimal medium. All experiments were performed in at least three replicates.

Supplementary Figure 8

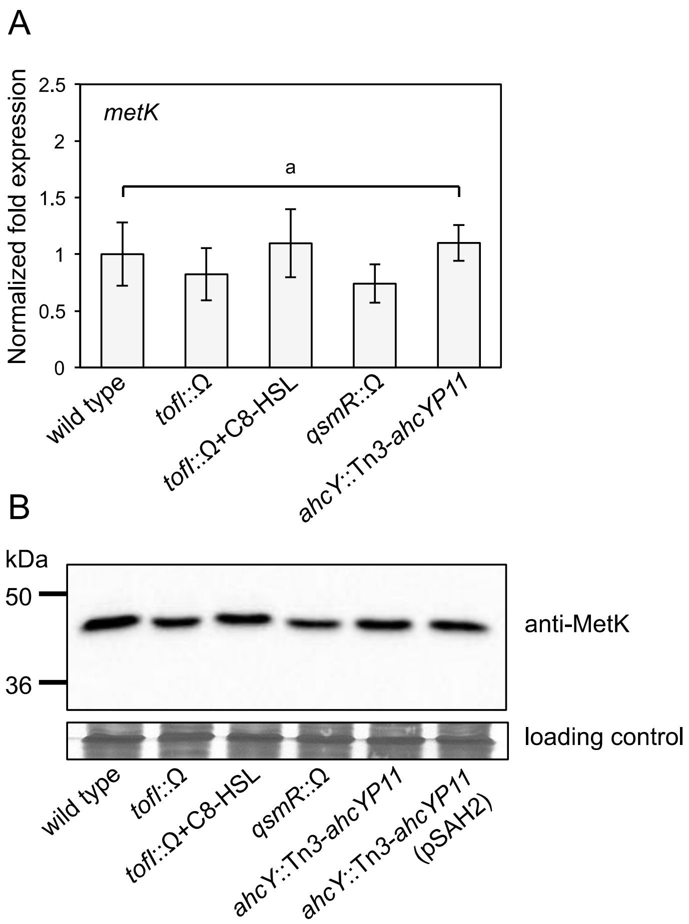

Regulation of SAM synthase encoded by *metK* in BAH2. The strains used for this analysis were BGR1 (wild type), BGS2 (BGR1 *tofI*::Ω), BGS9 (BGR1 *qsmR*::Ω), and BAH2 (BGR1 *ahcY*::Tn*3-ahcYP11*). The plasmid pSAH2 carries a 3.3-kb fragment with a putative promoter region and *ahcY* gene in pLAFR3. (**A**) Expression of *metK* in *B. glumae* strains. Gene expression levels after 10 h of incubation were quantified by qRT-PCR with three biological replicates. The letters above each mean represent groupings of statistical significance based on ANOVA/Tukey's correction for multiple comparisons. A value of $p < 0.05$ represents significant differences among strains. (**B**) Immunoblot analysis of MetK using an anti-SAM synthase antibody. The image of the blot stained with Coomassie brilliant blue R-250 (loading control) shows equal loading of samples in all lanes.
